# Efficacy and safety of tofacitinib in patients with rheumatoid arthritis by previous treatment: post hoc analysis of phase II/III trials

**DOI:** 10.1186/s13075-023-03154-z

**Published:** 2023-11-02

**Authors:** John Tesser, Ahmet Gül, Ewa Olech, Kurt Oelke, Tatjana Lukic, Kenneth Kwok, Abbas Ebrahim

**Affiliations:** 1 Arizona Arthritis & Rheumatology Research, Arizona Arthritis & Rheumatology Associates, Glendale, AZ USA; 2 Department of Internal Medicine, Division of Rheumatology, Istanbul Faculty of Medicine, Istanbul University, Istanbul, Turkey; 3 UNLV School of Medicine, Las Vegas, NV USA; 4 Rheumatic Disease Center, Glendale, WI USA; 5 Inflammation & Immunology, Pfizer Inc, New York, NY USA; 6 Inflammation & Immunology, Pfizer Inc, 500 Arcola Road, Collegeville, PA 19426 USA

**Keywords:** Antirheumatic agents, Arthritis, Methotrexate

## Abstract

**Background:**

This study sought to evaluate the efficacy and safety of tofacitinib in patients with rheumatoid arthritis with distinct treatment histories.

**Methods:**

Pooled phase II/III trial data from patients who received tofacitinib 5 or 10 mg twice daily or placebo, as monotherapy or with conventional synthetic (cs) disease-modifying antirheumatic drugs (DMARDs), were analyzed post hoc. Separate evaluations were conducted for populations with a prior inadequate response (IR) to: 1) non-methotrexate (MTX) csDMARDs only (non-MTX csDMARD-IR; *n* = 537); 2) MTX (MTX-IR; *n* = 3113); and 3) biologic (b)DMARDs (bDMARD-IR; *n* = 782). Efficacy outcomes included rates of response (American College of Rheumatology 20/50/70% response criteria) and remission (Disease Activity Score in 28 joints derived from 4 measures, erythrocyte sedimentation rate [DAS28-4(ESR)] < 2.6) at month 3, and changes from baseline in DAS28-4(ESR) and Health Assessment Questionnaire–Disability Index scores. Safety was assessed up to month 24.

**Results:**

At month 3, efficacy was generally improved with tofacitinib (both doses) vs placebo in each population. Generally, efficacy outcomes with tofacitinib were numerically more favorable in non-MTX csDMARD-IR vs MTX-IR or bDMARD-IR patients. Over 24 months, crude incidence rates for adverse events (AEs), serious AEs, and discontinuations due to AEs were generally numerically lower in non-MTX csDMARD-IR and MTX-IR vs bDMARD-IR populations; rates for AEs of special interest were generally similar across populations.

**Conclusions:**

Tofacitinib provided clinical benefit across patients with rheumatoid arthritis with a range of prior treatment experience but may have greater efficacy and an improved benefit/risk profile in those with fewer prior treatments.

**Trial registration:**

NCT00147498/NCT00413660/NCT00550446/NCT00603512/NCT00687193/NCT00976599/NCT01359150/NCT00847613/NCT00814307/NCT00853385/NCT00960440/NCT01039688/NCT00856544.

**Supplementary Information:**

The online version contains supplementary material available at 10.1186/s13075-023-03154-z.

## Background

Rheumatoid arthritis (RA) is an autoimmune disease characterized by systemic inflammation, persistent synovitis, and, potentially, joint destruction [1]. Treatments attempt to achieve sustained remission or low disease activity [2]. To this end, a range of therapies are available, including conventional synthetic (cs) disease-modifying antirheumatic drugs (DMARDs), biologic (b)DMARDs, and targeted synthetic (ts)DMARDs [2, 3]. Therapies are considered based on disease activity, comorbidities, and other risk factors, in the context of patient preferences, and have different mechanisms of action to address the heterogeneity of RA [2, 3].

Patients with active RA generally receive csDMARDs, usually methotrexate (MTX), as first-line therapy [2, 3], unless they have low disease activity, in which case first-line hydroxychloroquine or sulfasalazine have recently been conditionally recommended over MTX [3]. However, in clinical practice, contraindications can result in not all patients being candidates for treatment with MTX, while other patients may exhibit poor adherence to or may be intolerant of MTX therapy. For these patients, treatment with non-MTX csDMARDs, particularly sulfasalazine or leflunomide, is recommended [2]. Those with an inadequate response (IR) to MTX and/or non-MTX csDMARDs may receive treatment with bDMARDs (e.g., tumor necrosis factor inhibitors [TNFi]) or tsDMARDs (e.g., tofacitinib or other Janus kinase [JAK] inhibitors), preferably in combination with csDMARDs [2, 3].

The efficacy and safety of tofacitinib 5 and 10 mg twice daily (BID), administered as monotherapy or in combination with csDMARDs (mainly MTX) in patients with moderately to severely active RA have been demonstrated in phase II [4–8], phase III [9–15], and phase IIIb/IV [16, 17] randomized controlled trials with up to 72 months of follow-up, and in long-term extension studies with up to 114 months of observation [18–20]. In general, patients enrolled in these trials had experienced treatment failure with or had an IR to ≥ 1 csDMARD or bDMARD.

In this post hoc analysis, we evaluated the efficacy and safety of tofacitinib in three distinct populations of patients with RA based on prior line of therapy: those with an IR to non-MTX csDMARDs; those with an IR to MTX; and those with an IR to bDMARDs.

## Methods

### Design and patient populations

This was a post hoc analysis of pooled data from seven phase II (NCT00147498, NCT00413660, NCT00550446, NCT00603512, NCT00687193, NCT00976599, and NCT01359150) and six phase III (ORAL Scan [NCT00847613], ORAL Solo [NCT00814307], ORAL Standard [NCT00853385], ORAL Step [NCT00960440], ORAL Start [NCT01039688], and ORAL Sync [NCT00856544]) randomized, double-blind trials of tofacitinib in patients with RA. Full study design details have been published previously [4–13, 15, 21, 22] and are summarized in Supplemental Table 1 (see Additional file 1).

The trials enrolled patients aged ≥ 18 years with a diagnosis of active RA according to the American College of Rheumatology (ACR) 1987 Revised Criteria [23]. Patients had a previous IR to ≥ 1 DMARD [8], specifically a csDMARD [4–7, 9, 11, 12, 14, 15, 21], or bDMARD [7, 9, 12] (TNFi [4, 10]).

This post hoc analysis included data from patients who had received tofacitinib 5 or 10 mg BID, or placebo, as monotherapy or in combination with csDMARDs, in these trials. Although 10 mg BID is not the widely approved tofacitinib dose for the treatment of RA, it was included in this analysis for completeness. This analysis did not include studies that evaluated the 11 mg once-daily extended-release formulation of tofacitinib (e.g., ORAL Shift [NCT02831855]; NCT02281552). Separate evaluations were conducted for populations with a prior IR to: 1) non-MTX csDMARDs only (non-MTX csDMARD-IR); 2) MTX (MTX-IR; and 3) bDMARDs (bDMARD-IR). Patients were assigned to an appropriate analysis cohort based on the therapy they had received prior to tofacitinib, regardless of the trial in which they had participated. For the purposes of this analysis, IR included both intolerance and an incomplete response.

Each trial was conducted in accordance with the Declaration of Helsinki and International Council for Harmonisation Guidelines for Good Clinical Practice and approved by the institutional review board and/or independent ethics committee for each study center. All patients provided written informed consent.

### Outcomes

Efficacy outcomes were analyzed at month 3 (the latest placebo-controlled time point common across studies; trials of < 3 months’ duration were excluded from efficacy analyses) and included: proportion of patients achieving an improvement of ≥ 20%, ≥ 50%, or ≥ 70% in ACR response criteria (ACR20, ACR50, and ACR70 response); proportion of patients achieving remission, defined as a Disease Activity Score in 28 joints derived from 4 measures, erythrocyte sedimentation rate (DAS28-4[ESR]) of < 2.6; change from baseline in DAS28-4(ESR); and change from baseline in Health Assessment Questionnaire-Disability Index (HAQ-DI) score. Within the bDMARD-IR cohort, efficacy outcomes were also stratified by the number of failed bDMARDs (1 or ≥ 2).

Safety outcomes were reported through month 24 and included treatment-emergent adverse events (AEs), serious AEs (SAEs), discontinuations due to AEs, and AEs of special interest (AESIs). An SAE was defined as any untoward medical occurrence at any dose that resulted in death, was life-threatening, required inpatient hospitalization or prolongation of hospitalization, or resulted in persistent or significant disability/incapacity or a congenital anomaly/birth defect. AESIs included serious infection events, opportunistic infections (excluding tuberculosis), tuberculosis, herpes zoster (non-serious and serious), major adverse cardiovascular events, malignancies (excluding non-melanoma skin cancer), deep vein thrombosis, pulmonary embolism, and death.

### Statistical analysis

Efficacy and safety outcomes were assessed in the full analysis set, defined as all patients who were randomized and received ≥ 1 dose of the study drug. For efficacy analyses, binary endpoints (e.g., ACR20 response) were compared between tofacitinib (5 or 10 mg BID) and placebo in a post hoc analysis by forming a z-score using the normal approximation to the binomial. Missing values were computed using the non-responder imputation method. Continuous endpoints (e.g., HAQ-DI score) were analyzed using a mixed-effect model for repeated (longitudinal) measurement with no imputation for missing data. The fixed effects of treatment, visit, treatment-by-visit interaction, geographic region, and respective baseline score were included, with patient as a random effect.

For AEs, SAEs, discontinuations due to AEs, and AESIs, crude incidence rates (CIRs; unique patients with events/100 patient-years) were calculated based on the full duration of each study (up to 24 months). An exact Poisson 95% confidence interval (CI) adjusted for exposure time was calculated for each CIR. The proportions of patients with events were also calculated for these and other AEs. All analyses are descriptive and were based on observed cases without any imputation.

## Results

### Patients

**Fig. 1:**
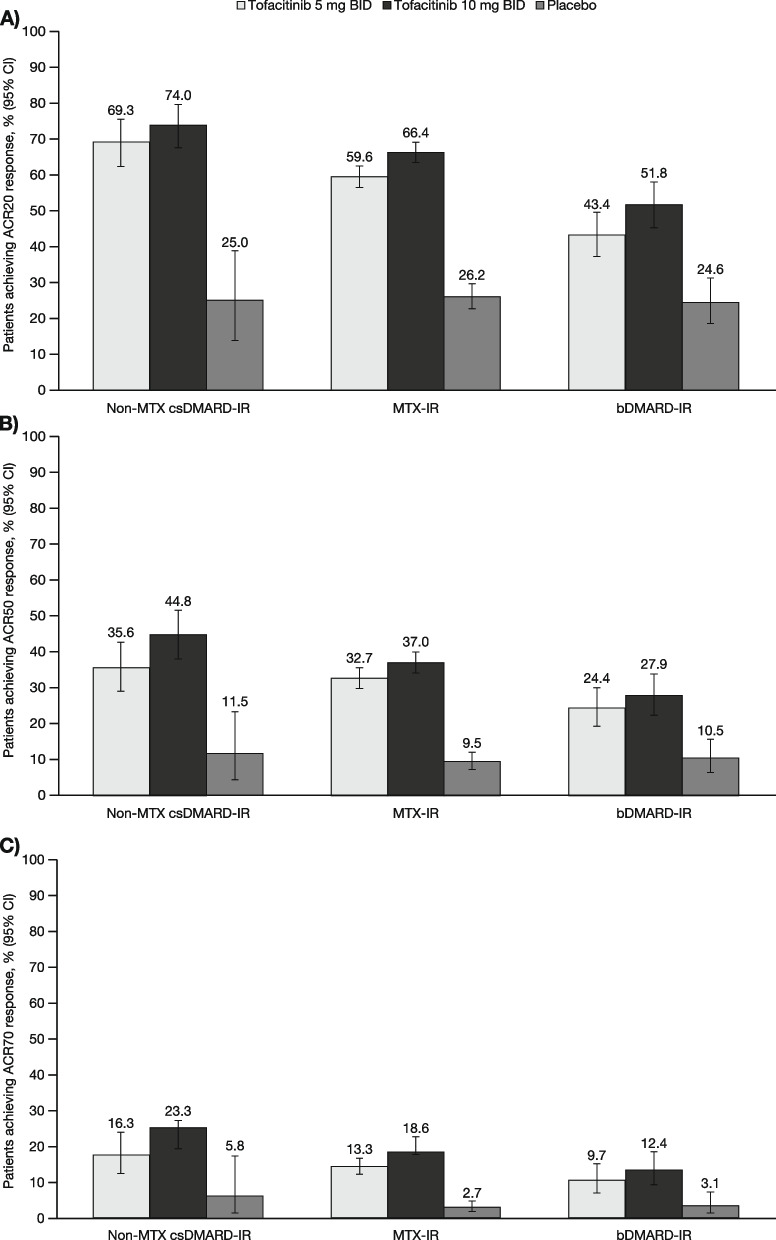
Proportion (95% CI) of patients achieving ACR responses at month 3. The plots show (**A**) ACR20, (**B**) ACR50, and (**C**) ACR70 response rates. Data presented for the FAS; non responder imputation. Non-MTX csDMARD-IR (i.e., non-MTX csDMARD-IR but not bDMARD-IR) FAS: tofacitinib 5 mg BID, *n* = 202; tofacitinib 10 mg BID, *n* = 219; placebo, *n* = 52. MTX-IR (i.e., MTX-IR but not bDMARD-IR) FAS: tofacitinib 5 mg BID, *n* = 1072; tofacitinib 10 mg BID, *n* = 1102; placebo, *n* = 631. bDMARD-IR FAS: tofacitinib 5 mg BID, *n* = 258; tofacitinib 10 mg BID, *n* = 251; placebo, *n* = 191. ACR20/50/70 American College of Rheumatology ≥ 20/50/70% response criteria, bDMARD biologic disease-modifying antirheumatic drug, BID twice daily, CI confidence interval, csDMARD conventional synthetic disease-modifying antirheumatic drug, FAS full analysis set, IR inadequate response or intolerance, MTX methotrexate

**Fig. 2:**
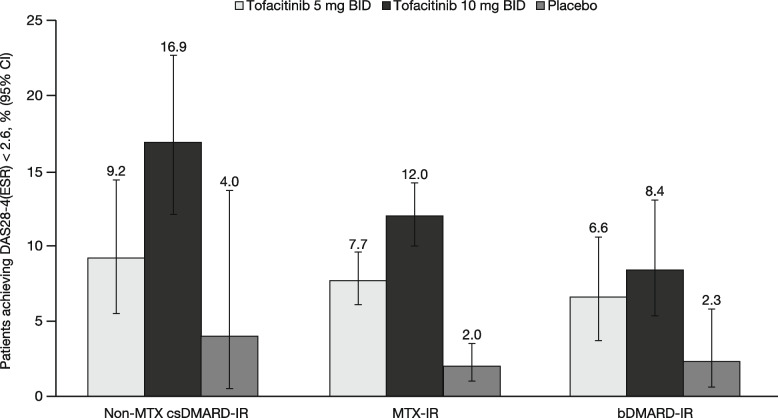
Proportion (95% CI) of patients achieving DAS28-4(ESR)-defined remission at month 3. Remission was defined as DAS28-4-(ESR) < 2.6. Data presented for the FAS; non responder imputation. Non-MTX csDMARD-IR (i.e., non-MTX csDMARD-IR but not bDMARD-IR) FAS: tofacitinib 5 mg BID, *n* = 184; tofacitinib 10 mg BID, *n* = 207; placebo, *n* = 50. MTX-IR (i.e., MTX-IR but not bDMARD-IR) FAS: tofacitinib 5 mg BID, *n* = 948; tofacitinib 10 mg BID, *n* = 969; placebo, *n* = 564. bDMARD-IR FAS: tofacitinib 5 mg BID, *n* = 229; tofacitinib 10 mg BID, *n* = 225; placebo, *n* = 175. bDMARD biologic disease-modifying antirheumatic drug, BID twice daily, CI confidence interval, csDMARD conventional synthetic disease-modifying antirheumatic drug, DAS28-4(ESR) Disease Activity Score in 28 joints derived from 4 measures, erythrocyte sedimentation rate, FAS full analysis set, IR inadequate response or intolerance, MTX methotrexate

**Fig. 3:**
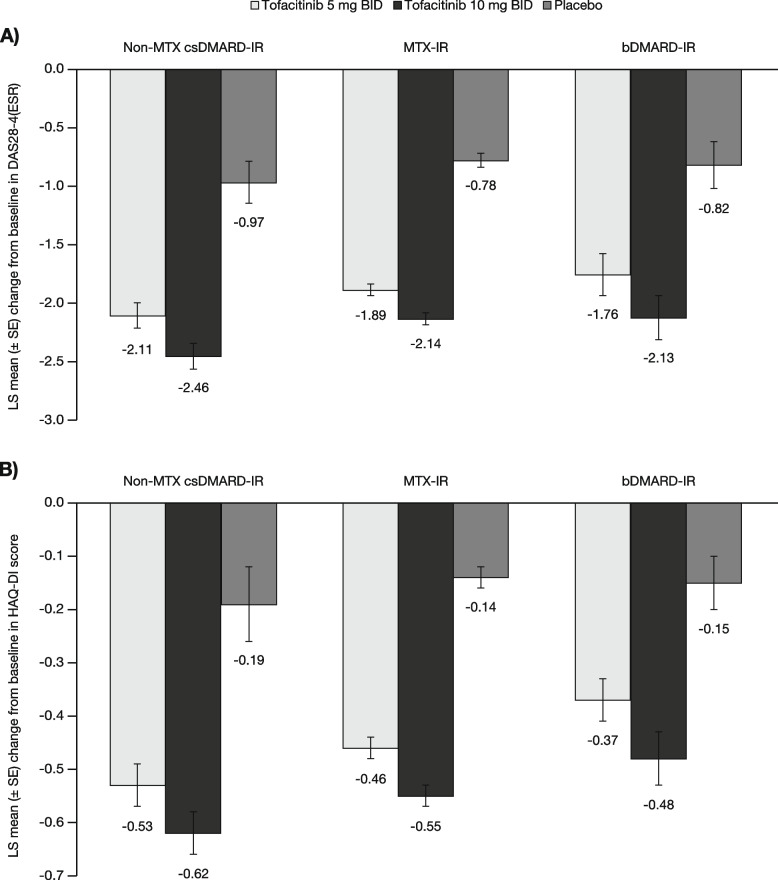
LS mean (SE) change from baseline at month 3 in (**A**) DAS28-4(ESR) and (**B**) HAQ-DI. Data presented for the FAS. Non-MTX csDMARD-IR (i.e., non-MTX csDMARD-IR but not bDMARD-IR) FAS for DAS28-4(ESR)/HAQ-DI: tofacitinib 5 mg BID, *n* = 176/196; tofacitinib 10 mg BID, *n* = 199/215; placebo, *n* = 46/48. MTX-IR (i.e., MTX-IR but not bDMARD-IR) FAS for DAS28-4(ESR)/HAQ-DI: tofacitinib 5 mg BID, *n* = 888/1019; tofacitinib 10 mg BID, *n* = 901/1041; placebo, *n* = 500/577. bDMARD-IR FAS for DAS28 4(ESR)/HAQ-DI: tofacitinib 5 mg BID, *n* = 213/236; tofacitinib 10 mg BID, *n* = 207/230; placebo, *n* = 156/169. bDMARD biologic disease modifying antirheumatic drug, BID twice daily, csDMARD conventional synthetic disease-modifying antirheumatic drug, DAS28-4(ESR) Disease Activity Score in 28 joints derived from 4 measures, erythrocyte sedimentation rate, FAS full analysis set, HAQ DI Health Assessment Questionnaire-Disability Index, IR inadequate response or intolerance, LS least squares, MTX methotrexate, SE standard error

A total of 4432 patients were included in this analysis, with the non-MTX csDMARD-IR, MTX-IR and bDMARD-IR populations comprising 537 (tofacitinib 5 mg BID, *n* = 208; tofacitinib 10 mg BID, *n* = 247; placebo, *n* = 82), 3113 (tofacitinib 5 mg BID, *n* = 1147; tofacitinib 10 mg BID, *n* = 1192; placebo, *n* = 774), and 782 (tofacitinib 5 mg BID, *n* = 270; tofacitinib 10 mg BID, *n* = 289; placebo, *n* = 223) patients, respectively. The number of patients in the full analysis of each patient population differed across the various efficacy outcomes, as shown in Figs. 1, 2 and 3.

Patient demographics and baseline disease characteristics were generally similar across the populations, with the mean age ranging from 49.7 to 54.6 years, the majority of patients being female (80.3–85.4%), and the mean DAS28-4(ESR) score ranging from 6.2 to 6.5 (Table 1). The median duration of RA was shorter for csDMARD groups (non-MTX csDMARDs-IR, 2.0–4.5 years; MTX-IR 5.6–6.0 years) than for the bDMARD-IR population (9.8–10.8 years). There were also noticeable differences among the populations in the proportions of patients who were taking concomitant corticosteroids at baseline, with proportions (across the tofacitinib 5 mg BID, tofacitinib 10 mg BID, and placebo groups) of 52.4–63.5% in the non-MTX csDMARD-IR population, 64.8–67.6% in the MTX-IR population and 72.2–75.1% in the bDMARD-IR population. Data for the individual treatment groups are reported in Table 1.

**Table 1:** Patient demographics, baseline disease characteristics, and prior DMARD exposure

	**Tofacitinib 5 mg BID**			**Tofacitinib 10 mg BID**			**Placebo**		
	**Non-MTX csDMARD-IR**^**a**^** (*n* = 208)**	**MTX-IR**^**b**^** (*n* = 1147)**	**bDMARD-IR (*n* = 270)**	**Non-MTX csDMARD-IR**^**a**^** (*n* = 247)**	**MTX-IR**^**b**^** (*n* = 1192)**	**bDMARD-IR (*n* = 289)**	**Non-MTX csDMARD-IR**^**a**^** (*n* = 82)**	**MTX-IR**^**b**^** (*n* = 774)**	**bDMARD-IR (*n* = 223)**
Age, years									
Mean (SD)	50.3 (11.9)	52.5 (11.8)	54.6 (11.1)	50.7 (12.4)	52.3 (11.6)	54.6 (10.8)	49.7 (11.7)	52.3 (12.1)	53.7 (12.0)
Median (range)	51.0 (18–76)	53.0 (18–86)	55.0 (20–83)	51.0 (18–80)	53.0 (18–85)	56.0 (21–84)	50.0 (18–73)	54.0 (18–81)	54.0 (20–82)
Female, n (%)	167 (80.3)	967 (84.3)	227 (84.1)	211 (85.4)	1004 (84.2)	236 (81.7)	69 (84.1)	638 (82.4)	179 (80.3)
RA duration, years									
Mean (SD)	5.2 (7.1)	8.1 (7.5)	12.0 (8.9)	6.7 (7.7)	8.1 (7.8)	12.2 (8.5)	7.2 (7.7)	8.6 (8.2)	11.6 (8.8)
Median (range)	2.0 (0.0–34.0)	5.6 (0.1–46.0)	10.0 (0.9–55.0)	3.3 (0.1–34.0)	5.6 (0.1–49.0)	10.8 (0.4–45.0)	4.5 (0.1–36.0)	6.0 (0.3–49.4)	9.8 (0.3–47.0)
DAS28-4(ESR), mean (SD)	6.5 (0.9)	6.4 (1.0)	6.5 (1.0)	6.4 (0.9)	6.4 (1.0)	6.4 (1.0)	6.2 (0.8)	6.3 (1.0)	6.4 (1.1)
HAQ-DI score, mean (SD)	1.5 (0.6)	1.4 (0.7)	1.6 (0.6)	1.4 (0.7)	1.4 (0.7)	1.5 (0.6)	1.4 (0.7)	1.4 (0.7)	1.6 (0.6)
Concomitant corticosteroid use, n (%)	132 (63.5)	775 (67.6)	196 (72.6)	150 (60.7)	773 (64.8)	217 (75.1)	43 (52.4)	508 (65.6)	161 (72.2)
Corticosteroid dose, mg									
Mean (SD)	4.0 (3.9)	4.0 (3.9)	4.5 (4.1)	4.6 (13.9)	3.9 (4.0)	4.4 (3.9)	3.1 (4.1)	3.7 (3.8)	4.6 (4.0)
Prior treatment with specific csDMARDs, n (%)									
Chloroquine	41 (19.7)	165 (14.4)	9 (3.3)	47 (19.0)	163 (13.7)	16 (5.5)	7 (8.5)	103 (13.3)	7 (3.1)
Hydroxychloroquine	78 (37.5)	255 (22.2)	54 (20.0)	101 (40.9)	271 (22.7)	63 (21.8)	21 (25.6)	165 (21.3)	38 (17.0)
Leflunomide	45 (21.6)	256 (22.3)	70 (25.9)	46 (18.6)	237 (19.9)	75 (26.0)	13 (15.9)	146 (18.9)	44 (19.7)
MTX	13 (6.3)	1147 (100.0)	260 (96.3)	17 (6.9)	1192 (100.0)	271 (93.8)	9 (11.0)	774 (100.0)	216 (96.9)
Sulfasalazine	69 (33.2)	289 (25.2)	52 (19.3)	79 (32.0)	333 (27.9)	55 (19.0)	19 (23.2)	212 (27.4)	34 (15.3)
Other	14 (6.7)	131 (11.4)	29 (10.7)	18 (7.3)	114 (9.6)	36 (12.5)	11 (13.4)	88 (11.4)	16 (7.2)
Number of failed bDMARDs, n (%)									
1	NA	NA	168 (62.2)	NA	NA	183 (63.3)	NA	NA	137 (61.4)
2	NA	NA	81 (30.0)	NA	NA	65 (22.5)	NA	NA	66 (29.6)
≥ 3	NA	NA	21 (7.8)	NA	NA	41 (14.2)	NA	NA	20 (9.0)
Type of failed bDMARD, n (%)									
TNFi	NA	NA	234 (86.7)	NA	NA	244 (84.4)	NA	NA	195 (87.4)
Non-TNFi	NA	NA	15 (5.6)	NA	NA	8 (2.8)	NA	NA	10 (4.5)
Both TNFi and non-TNFi	NA	NA	21 (7.8)	NA	NA	37 (12.8)	NA	NA	18 (8.1)
RA duration in patients with bDMARD failure, years									
1 failed bDMARD									
Mean (SD)	NA	NA	11.6 (8.5)	NA	NA	11.8 (8.3)	NA	NA	11.1 (8.7)
Median (range)	NA	NA	9.7 (1.0–38.0)	NA	NA	10.6 (0.6–40.0)	NA	NA	9.0 (0.3–47.0)
≥ 2 failed bDMARDs									
Mean (SD)	NA	NA	12.6 (9.6)	NA	NA	12.7 (8.9)	NA	NA	12.4 (9.0)
Median (range)	NA	NA	10.3 (0.9–55.0)	NA	NA	11.0 (0.4–45.0)	NA	NA	11.0 (1.1–39.0)

Data presented for the FAS

*Abbreviations: bDMARD* biologic disease-modifying antirheumatic drug, *BID* twice daily, *csDMARD* conventional synthetic disease-modifying antirheumatic drug, *DAS28-4(ESR)* Disease Activity Score in 28 joints derived from 4 measures, erythrocyte sedimentation rate, *DMARD* disease-modifying antirheumatic drug, *HAQ-DI* Health Assessment Questionnaire-Disability Index, *IR* inadequate response or intolerance, *MTX* methotrexate, *NA* not applicable, *RA* rheumatoid arthritis, *SD* standard deviation, *TNFi* tumor necrosis factor inhibitors

^a^Non-MTX csDMARD-IR but not bDMARD-IR

^b^MTX-IR but not bDMARD-IR

The most commonly used prior csDMARDs in the non-MTX csDMARD-IR population were hydroxychloroquine (25.6–40.9% of patients across treatment groups) and sulfasalazine (23.2–33.2%) (Table 1). Although some patients (6.3–11.0%) in this population had previously received MTX, they were not MTX-IR.

Similarly, in the MTX-IR population, the most commonly used prior csDMARDs (other than MTX) included hydroxychloroquine (21.3–22.7% of patients), sulfasalazine (25.2–27.9%), and leflunomide (18.9–22.3%). In the bDMARD-IR population, most patients (93.8–96.9%) had previously received MTX treatment and 61.4–63.3% had failed just 1 bDMARD, with the longest median RA durations being observed in patients who had failed ≥ 2 bDMARDs (Table 1).

### Efficacy

In all three patient populations (non-MTX csDMARD-IR, MTX-IR, and bDMARD-IR), rates of ACR20, ACR50, and ACR70 response at month 3 were numerically higher with tofacitinib 5 and 10 mg BID than with placebo, although the 95% CIs overlapped between tofacitinib 5 mg BID and placebo in the non-MTX csDMARD-IR and bDMARD-IR populations for ACR70 (Fig. 1). Response rates were numerically lower with tofacitinib 5 vs 10 mg BID, although the 95% CIs generally overlapped, except for ACR20 and ACR70 response in the MTX-IR population (Fig. 1). Compared with the MTX-IR and bDMARD-IR populations, the non-MTX csDMARD-IR population had numerically higher proportions of patients achieving an ACR20, ACR50 or ACR70 response within each treatment group (Fig. 1). The 95% CIs for these differences generally overlapped between all three patient populations, except for ACR20 comparisons between the non-MTX csDMARD-IR and bDMARD-IR groups for tofacitinib 5 or 10 mg BID.

Similarly, rates of DAS28-4(ESR)-defined remission were numerically higher with tofacitinib 5 and 10 mg BID than placebo at month 3 in the non-MTX csDMARD-IR and bDMARD-IR populations (although 95% CIs overlapped); DAS28-4(ESR)-defined remission rates were higher with tofacitinib 5 and 10 mg BID than placebo at month 3 in the MTX-IR population (Fig. 2). The proportions of patients who achieved remission were numerically greater with tofacitinib 10 vs 5 mg BID, although the 95% CIs overlapped in all but the MTX-IR population (Fig. 2). Regardless of treatment group, the rate of DAS28-4(ESR)-defined remission was numerically higher in the non-MTX csDMARD-IR population than in the MTX-IR or bDMARD-IR populations, although the 95% CIs overlapped (Fig. 2).

Consistent with these findings, there were greater least squares (LS) mean reductions from baseline in DAS28-4(ESR) and HAQ-DI scores at month 3 with tofacitinib 5 and 10 mg BID vs placebo in all populations (Fig. 3). The LS mean reductions in these scores were numerically greater with tofacitinib 10 vs 5 mg BID, with mostly non-overlapping or touching 95% CIs. Regardless of the tofacitinib dose, reductions from baseline in DAS28-4(ESR) scores were numerically greater in the non-MTX csDMARD-IR population than in the MTX-IR or bDMARD-IR populations. Reductions from baseline in HAQ-DI score were numerically greater with each tofacitinib dose in the non-MTX csDMARD-IR and MTX-IR populations vs the bDMARD population (Fig. 3).

When the bDMARD-IR population was stratified by number of failed prior bDMARDs (1 or ≥ 2), generally similar proportions of patients achieved an ACR20/50/70 response and DAS28-4(ESR) remission at month 3. The proportions of patients with ≥ 2 prior failed bDMARDs achieving an ACR20 and ACR50 response were greater and numerically greater (95% CIs overlapped), respectively, with both tofacitinib doses compared with placebo. Mean reductions from baseline in HAQ-DI score at month 3 were similar regardless of number of prior failed bDMARDs (see Supplemental Fig. 1 in Additional file 2). 

### Safety

Across the patient populations, the majority of patients treated with tofacitinib 5 mg BID (70.2–74.4%) or tofacitinib 10 mg BID (70.4–72.7%) reported AEs over 24 months, compared with 42.7–58.7% of patients treated with placebo. In the respective treatment groups, 7.8–10.6%, 6.9–8.6%, and 2.4–4.5% of patients experienced SAEs and 8.5–9.3%, 8.5–9.2%, and 2.4–4.5% discontinued treatment due to AEs (Table 2).

**Table 2:** Incidence of TEAEs, SAEs, discontinuations due to AEs, and AESIs occurring up to month 24

	**Tofacitinib 5 mg BID**			**Tofacitinib 10 mg BID**			**Placebo**		
	**Non-MTX csDMARD-IR**^**a**^** (*n* = 208)**	**MTX-IR**^**b**^** (*n* = 1147)**	**bDMARD-IR (*n* = 270)**	**Non-MTX csDMARD-IR**^**a**^** (*n* = 247)**	**MTX-IR**^**b**^** (*n* = 1192)**	**bDMARD-IR (*n* = 289)**	**Non-MTX csDMARD-IR**^**a**^** (*n* = 82)**	**MTX-IR**^**b**^** (*n* = 774)**	**bDMARD-IR (*n* = 223)**
TEAEs									
n (%)	146 (70.2)	825 (71.9)	201 (74.4)	174 (70.4)	860 (72.1)	210 (72.7)	35 (42.7)	414 (53.5)	131 (58.7)
CIR^c^ (95% CI)	118.4 (99.9–139.2)	190.4 (177.6–203.8)	311.0 (269.5–357.1)	136.3 (116.8–158.1)	193.5 (180.7–206.8)	371.9 (323.3–425.7)	264.1 (183.9–367.3)	294.9 (267.2–324.7)	429.5 (359.1–509.7)
SAEs									
n (%)	21 (10.1)	122 (10.6)	21 (7.8)	21 (8.5)	103 (8.6)	20 (6.9)	2 (2.4)	25 (3.2)	10 (4.5)
CIR^c^ (95% CI)	8.5 (5.2–12.9)	12.5 (10.4–14.9)	12.6 (7.8–19.3)	7.4 (4.6–11.3)	9.9 (8.1–12.0)	12.5 (7.6–19.3)	10.8 (1.3–39.1)	11.6 (7.5–17.1)	20.5 (9.8–37.7)
Discontinuation due to AEs									
n (%)	18 (8.7)	97 (8.5)	25 (9.3)	21 (8.5)	110 (9.2)	25 (8.7)	2 (2.4)	26 (3.4)	10 (4.5)
CIR^c^ (95% CI)	7.1 (4.2–11.2)	9.6 (7.8–11.7)	14.4 (9.3–21.2)	7.2 (4.5–11.0)	10.3 (8.5–12.4)	15.3 (9.9–22.6)	10.8 (1.3–39.1)	12.0 (7.8–17.5)	20.4 (9.8–37.6)
AESIs									
Serious infection event									
n (%)	7 (3.4)	35 (3.1)	3 (1.1)	4 (1.6)	39 (3.3)	5 (1.7)	1 (1.2)	3 (0.4)	2 (0.9)
CIR^c^ (95% CI)	2.8 (1.1–5.7)	3.4 (2.4–4.8)	1.7 (0.4–5.0)	1.4 (0.4–3.5)	3.6 (2.6–5.0)	3.0 (1.0–7.1)	5.4 (0.1–30.1)	1.4 (0.3–4.0)	4.1 (0.5–14.7)
Opportunistic infection, excluding tuberculosis									
n (%)	1 (0.5)	1 (0.1)	0 (0.0)	0 (0.0)	6 (0.5)	0 (0.0)	0 (0.0)	0 (0.0)	0 (0.0)
CIR^c^ (95% CI)	0.4 (0.0–2.2)	0.1 (0.0–0.6)	0.0 (0.0–2.1)	0.0 (0.0–1.3)	0.6 (0.2–1.2)	0.0 (0.0–2.3)	0.0 (0.0–19.9)	0.0 (0.0–1.7)	0.0 (0.0–7.5)
Tuberculosis									
n (%)	0 (0.0)	0 (0.0)	0 (0.0)	1 (0.4)	7 (0.6)	0 (0.0)	0 (0.0)	0 (0.0)	0 (0.0)
CIR^c^ (95% CI)	0.0 (0.0–1.4)	0.0 (0.0–0.4)	0.0 (0.0–2.1)	0.3 (0.0–1.9)	0.7 (0.3–1.4)	0.0 (0.0–2.3)	0.0 (0.0–19.9)	0.0 (0.0–1.7)	0.0 (0.0–7.5)
Herpes zoster (non-serious and serious)									
n (%)	3 (1.4)	37 (3.2)	9 (3.3)	10 (4.0)	51 (4.3)	10 (3.5)	0 (0.0)	6 (0.8)	0 (0.0)
CIR^c^ (95% CI)	1.2 (0.2–3.5)	3.7 (2.6–5.1)	5.3 (2.4–10.0)	3.5 (1.7–6.4)	4.9 (3.6–6.4)	6.3 (3.0–11.6)	0.0 (0.0–19.9)	2.8 (1.0–6.0)	0.0 (0.0–7.5)
Major adverse cardiovascular events^d^									
n (%)	2 (1.1)	3 (0.3)	2 (0.8)	2 (1.0)	5 (0.5)	1 (0.4)	0 (0.0)	1 (0.2)	0 (0.0)
CIR^c^ (95% CI)	0.8 (0.1–2.9)	0.3 (0.1–0.9)	1.2 (0.1–4.2)	0.7 (0.1–2.6)	0.5 (0.2–1.2)	0.7 (0.0–3.6)	0.0 (0–41.1)	0.7 (0.0–3.7)	0.0 (0.0–8.7)
Malignancies (excluding non-melanoma skin cancer)									
n (%)	1 (0.5)	8 (0.7)	2 (0.7)	1 (0.4)	8 (0.7)	3 (1.0)	0 (0.0)	0 (0.0)	0 (0.0)
CIR^c^ (95% CI)	0.4 (0.0–2.2)	0.8 (0.3–1.5)	1.1 (0.1–4.1)	0.3 (0.0–1.9)	0.8 (0.3–1.5)	1.8 (0.4–5.4)	0.0 (0.0–19.9)	0.0 (0.0–1.7)	0.0 (0.0–7.5)
Deep vein thrombosis									
n (%)	4 (1.9)	6 (0.5)	2 (0.7)	1 (0.4)	9 (0.8)	2 (0.7)	0 (0.0)	0 (0.0)	1 (0.4)
CIR^c^ (95% CI)	1.6 (0.4–4.0)	0.6 (0.2–1.3)	1.1 (0.1–4.1)	0.3 (0.0–1.9)	0.8 (0.4–1.6)	1.2 (0.2–4.4)	0.0 (0.0–19.9)	0.0 (0.0–1.7)	2.0 (0.1–11.3)
Pulmonary embolism									
n (%)	0 (0.0)	7 (0.6)	1 (0.4)	0 (0.0)	7 (0.6)	3 (1.0)	0 (0.0)	1 (0.1)	2 (0.9)
CIR^c^ (95% CI)	0.0 (0.00–1.4)	0.7 (0.3–1.4)	0.6 (0.0–3.2)	0.0 (0.0–1.3)	0.7 (0.3–1.3)	1.8 (0.4–5.4)	0.0 (0.0–19.9)	0.5 (0.0–2.6)	4.1 (0.5–14.7)
Deep vein thrombosis or pulmonary embolism									
n (%)	4 (1.9)	12 (1.0)	2 (0.7)	1 (0.4)	15 (1.3)	5 (1.7)	0 (0.0)	1 (0.1)	2 (0.9)
CIR^c^ (95% CI)	1.6 (0.4–4.0)	1.2 (0.6–2.1)	1.1 (0.1–4.1)	0.3 (0.0–1.9)	1.4 (0.8–2.3)	3.1 (1.0–7.1)	0.0 (0.0–19.9)	0.5 (0.0–2.6)	4.1 (0.5–14.7)
Deep vein thrombosis and pulmonary embolism									
n (%)	0 (0.0)	1 (0.1)	1 (0.4)	0 (0.0)	1 (0.1)	0 (0.0)	0 (0.0)	0 (0.0)	1 (0.4)
CIR^c^ (95% CI)	0.0 (0.0–1.4)	0.1 (0.0–0.6)	0.6 (0.0–3.2)	0.0 (0.0–1.3)	0.1 (0.0–0.5)	0.0 (0.0–2.3)	0.0 (0.0–19.9)	0.0 (0.0–1.7)	2.0 (0.1–11.3)
Death									
n (%)	1 (0.5)	9 (0.8)	2 (0.7)	1 (0.4)	3 (0.3)	0 (0.0)	0 (0.0)	1 (0.1)	0 (0.0)
CIR^c^ (95% CI)	0.4 (0.0–2.2)	0.9 (0.4–1.7)	1.1 (0.1–4.1)	0.3 (0.0–1.9)	0.3 (0.1–0.8)	0.0 (0.0–2.3)	0.0 (0.0–19.9)	0.5 (0.0–2.6)	0.0 (0.0–7.5)

Data presented for the FAS; non-responder imputation

*Abbreviations: AE* adverse event, *AESI* adverse event of special interest, *bDMARD* biologic disease-modifying antirheumatic drug, *BID* twice daily, *CI* confidence interval, *CIR* crude incidence rate (unique patients with events/100 PY), *csDMARD* conventional synthetic disease-modifying antirheumatic drug, *FAS* full analysis set; *IR* inadequate response or intolerance, *MTX* methotrexate, *PY* patient-years, *SAE* serious adverse event, *TEAE* treatment-emergent adverse event

^a^Non-MTX csDMARD-IR but not bDMARD-IR

^b^MTX-IR but not bDMARD-IR

^c^Per 100 PY

^d^N for tofacitinib 5 mg BID: 185 (non-MTX csDMARD-IR), 933 (MTX-IR), and 247 (bDMARD-IR); N for tofacitinib 10 mg BID: 204 (non-MTX csDMARD-IR), 942 (MTX-IR), and 241 (bDMARD-IR); N for placebo: 32 (non-MTX csDMARD-IR), 468 (MTX-IR), and 181 (bDMARD-IR)

The CIRs for AEs, SAEs, and discontinuations due to AEs in each of the three patient populations tended to be numerically lower with tofacitinib 5 and 10 mg BID than with placebo (with the exception of SAEs with tofacitinib 5 mg BID in the MTX-IR population), although the 95% CIs generally overlapped (Table 2). There were no marked numeric differences in the CIRs for these events between the tofacitinib doses (overlapping 95% CIs). However, irrespective of treatment group, the CIRs for treatment-emergent AEs, SAEs, and discontinuations due to AEs were numerically lower in the non-MTX csDMARD-IR population than in the MTX-IR population, and in each of these populations than in the bDMARD-IR population, although the 95% CIs generally overlapped (Table 2).

The AESIs with the highest CIR were generally herpes zoster (non-serious and serious) in patients treated with tofacitinib 5 or 10 mg BID and serious infection events in patients who received placebo (Table 2). The highest CIRs for AESIs were generally reported with tofacitinib 10 mg BID and, regardless of treatment group, some numeric differences in the CIRs were evident across the three patient populations; however, 95% CIs were generally overlapping (Table 2).

## Discussion

Adequate responses to RA therapies are not achieved or sustained by all patients, leaving clinicians with the challenge of determining the subsequent treatment to which a patient may potentially respond. The findings of this post hoc analysis of data from phase II and III trials in patients with RA suggest that, overall, treatment with tofacitinib 5 or 10 mg BID is associated with improved clinical and patient-reported efficacy outcomes vs placebo at 3 months in non-MTX csDMARD-IR, MTX-IR, and bDMARD-IR patient populations. Efficacy outcomes with tofacitinib were generally numerically more favorable in the non-MTX csDMARD-IR population than in the MTX-IR or bDMARD-IR populations, regardless of the tofacitinib dose; however, lower patient numbers in specific populations (e.g., non-MTX csDMARD-IR) limited the statistical power to identify potential differences between populations. As the non-MTX csDMARD-IR population had the shortest RA duration, and was therefore likely to have the fewest prior treatments, this finding suggests that tofacitinib may be more efficacious when used in earlier lines of treatment. In addition, joint damage accrues over time in patients with RA [24], likely making efficacy measures with a damage component less sensitive to change in later vs earlier disease [25].

Indeed, longer disease duration was found to be associated with a reduced likelihood of treatment response in patients with RA in an analysis of 14 randomized controlled RA trials, the majority of which evaluated the use of MTX [26]. Similarly, an analysis of a large cohort of patients with RA from the Consortium of Rheumatology Researchers of North America (CORRONA) registry found a greater likelihood of remission in patients who initiated therapy with a TNFi or non-bDMARD earlier in the disease course [27]. Moreover, when the impact of disease duration on treatment outcomes was assessed in bDMARD-naive patients with RA who initiated the bDMARD abatacept in the CORRONA registry, the magnitude of improvement in outcomes was greater in patients with a shorter vs longer disease duration [28]. However, not all studies support these findings [29, 30].

Although European Alliance of Associations for Rheumatology (EULAR) and ACR treatment guidelines for RA have indicated that evidence for differential responses to therapy by disease duration alone is lacking, long durations of disease, and the failure of several csDMARDs, have previously been acknowledged by EULAR as key factors in response rate reductions in patients initiating bDMARDs or tsDMARDs as subsequent therapies [31]. However, persistent moderate or high disease activity despite csDMARD therapy and failing ≥ 2 csDMARDs are factors associated with poor prognosis, with the addition of a bDMARD (JAK inhibitor considered when pertinent risk factors are taken into account) recommended by EULAR in patients with poor prognostic factors who fail to achieve treatment targets with their first csDMARD [2, 3].

In this analysis, the safety profile of tofacitinib up to 24 months was generally similar across the three evaluated patient populations, while CIRs for AEs, SAEs, and discontinuations due to AEs were generally numerically lower in earlier lines of therapy (i.e., in the non-MTX csDMARD-IR and MTX-IR populations vs the bDMARD-IR population [95% CIs overlapped], in which patients had the longest RA duration). This suggests that there may be an association between RA duration and the risk of AEs with tofacitinib. However, this may be confounded by the fact that a lower proportion of patients in the non-MTX csDMARD-IR population were receiving concomitant corticosteroids at baseline vs patients in the bDMARD-IR population, particularly as corticosteroids have been associated with increased occurrence of AEs [32]. Lower concomitant corticosteroid use at baseline may also have reduced potential drug-drug interactions, and therefore the likelihood of AEs. However, it is worth noting that CIRs for AESIs with tofacitinib were generally similar across the three patient populations; these AEs included serious infection events, opportunistic infections (excluding tuberculosis), tuberculosis, herpes zoster (non-serious and serious), major adverse cardiovascular events, malignancies (excluding non-melanoma skin cancer), deep vein thrombosis, pulmonary embolism, and death. In addition, it should be noted that the risk of specific safety events, such as herpes zoster and serious infections, may be greater when tofacitinib is used in combination with csDMARDs vs monotherapy [33]. However, due to low patient numbers in specific populations, the analyses in this study were not stratified by tofacitinib regimen (i.e., tofacitinib in combination with csDMARDs, or as monotherapy).

We are not aware of any RA trials or analyses that have assessed DMARD efficacy or safety outcomes in a range of patient populations across numerous trials, similar to those assessed in the current analysis. While a range of patient populations has been investigated in previous trials of tofacitinib (csDMARD-IR, MTX-IR, TNFi-IR) [10, 12, 13, 15], tocilizumab (DMARD-IR, csDMARD-IR, MTX-IR, bDMARD-IR, TNFi-IR) [34–38], baricitinib (MTX-IR, bDMARD-IR, TNFi-IR) [39, 40], upadacitinib (csDMARD, MTX-IR, bDMARD-IR) [41–43], and sarilumab (MTX-IR, TNFi-IR) [44, 45]; these patient populations were all studied within individual trials, rather than across trials with stratification by patient populations, as presented here. Comparing patient populations from individual trials presents challenges in drawing conclusions due to differing designs. To the best of our knowledge, only one other analysis has evaluated the impact of prior treatment on the safety and efficacy of a JAK inhibitor in patients with RA in a clinical trial setting. This was an exploratory analysis of data from a phase III trial of baricitinib in patients with active RA despite csDMARD therapy and ≥ 1 TNFi [46]. Rates of AEs generally appeared to be somewhat higher in patients with more vs less extensive prior use of bDMARDs, although the clinical efficacy of baricitinib did not appear to be impacted by the number of prior bDMARDs or RA duration. However, the small number of patients included in the subgroups may have impacted these findings.

The impact of prior bDMARD failures on efficacy and safety outcomes in patients with RA receiving the interleukin-6 inhibitor tocilizumab has also been determined in an analysis of data from an observational cohort study [47]. The likelihood of tocilizumab being discontinued was 2.2-fold higher in patients who had failed ≥ 3 bDMARDs, compared with those naïve to bDMARDs, and 1.8-fold higher, compared with those who had failed ≥ 2 bDMARDs. AEs and ineffectiveness were the most common reasons for tocilizumab discontinuation, although there were no clear correlations between the rates of these discontinuations and the number of prior bDMARD failures. Interestingly, when responses to a newly initiated DMARD were assessed in patients with RA in the CORRONA database, the total number of DMARDs previously used was not among the factors found to be predictive of functional improvement (including shorter disease duration, higher baseline function, the addition of another DMARD during follow-up, and the frequency with which DMARDs were changed) [48].

Certain limitations require consideration when interpreting the current findings, including the post hoc nature of the analysis and the fact that both the non-MTX csDMARD-IR and bDMARD-IR populations were substantially smaller than the MTX-IR population; this significantly reduced the statistical power to detect noteworthy population differences. The lower patient numbers in some populations also precluded analyses of tofacitinib efficacy and safety stratified on the basis of RA duration, which would have been interesting to explore further. These descriptive post hoc findings require confirmation in a prospective study.

## Conclusions

In conclusion, this analysis suggests that tofacitinib provides clinical benefit across patients with a range of prior treatment experience (non-MTX csDMARD-IR, MTX-IR, and bDMARD-IR patient populations). Tofacitinib may, however, have greater efficacy and an improved benefit/risk profile in patients with shorter duration of disease and limited prior DMARD treatment experience, including those with intolerance, contraindications or poor adherence to MTX therapy.

### Supplementary Information

**Additional file 1:** **Supplemental Table 1.** Summary of the clinical trials included in the analysis.

**Additional file 2:** **Supplemental Fig. 1.** fficacy outcomes in the bDMARD-IR population stratified by 1 or ≥ 2 prior bDMARD failure as assessed by proportion (95% CI) of patients achieving (A) ACR20, (B) ACR50, and (C) ACR70 response, (D) DAS28-4(ESR)-defined remission (score < 2.6), and (E) LS mean (SE) change from baseline in HAQ-DI score at month 3.

## Data Availability

Upon request, and subject to review, Pfizer will provide the data that support the findings of this study. Subject to certain criteria, conditions, and exceptions, Pfizer may also provide access to the related individual de-identified participant data. See https://www.pfizer.com/science/clinical-trials/trial-data-and-results for more information.

## Abbreviations

ACR: American College of Rheumatology
AE: Adverse event
AESI: Adverse event of special interest
bDMARD: Biologic DMARD
BID: Twice daily
CI: Confidence interval
CIR: Crude incidence rate
csDMARD: Conventional synthetic DMARD
DAS28-4(ESR): Disease Activity Score in 28 joints derived from 4 measures, erythrocyte sedimentation rate
DMARD: Disease-modifying antirheumatic drug
EULAR: European Alliance of Associations for Rheumatology
FAS: Full analysis set
HAQ-DI: Health Assessment Questionnaire-Disability Index
IR: Inadequate response
JAK: Janus kinase
LS: Least squares
MTX: Methotrexate
PY: Patient-years
RA: Rheumatoid arthritis
SAE: Serious adverse event
SD: Standard deviation
SE: Standard error
TEAE: Treatment-emergent adverse event
TNFi: Tumor necrosis factor inhibitors
