# Supplementary material for: Efficacy and safety of tofacitinib in patients with rheumatoid arthritis by previous treatment: post hoc analysis of phase II/III trials
Source: Arthritis Res Ther. 2023 Nov 2;25:214. doi: 10.1186/s13075-023-03154-z (PMC10621211; doi:10.1186/s13075-023-03154-z)
Supplement: Supplementary file 1 — Additional file 1: Supplemental Table 1. Summary of the clinical trials included in the analysis. [file 13075_2023_3154_MOESM1_ESM.pdf]

Supplemental Table 1 Summary of the clinical trials included in the analysis

ClinicalTrials.gov identifier (trial acronym)	Trial phase	Patient population	Tofacitinib arm(s)	Control arm(s)	Total patients in tofacitinib arm(s), *n*	Total patients in control arm(s), *n*	Study duration	Distinctive features
NCT00147498[a] [1]	IIa	Active RA with inadequate response or intolerance to MTX or certain bDMARDs	5, 15, or 30 mg BID monotherapy	Placebo	199[b]	65[b]	6 weeks	Dose-ranging study; tofacitinib monotherapy
NCT00413660 [2]	IIb	Active RA with inadequate response to MTX	1, 3, 5, 10, or 15 mg BID, or 20 mg QD with background MTX (patients receiving 1 mg BID, 3 mg BID, or 20 mg QD who were non-responders at month 3 were advanced to 5 mg BID)	Placebo with background MTX (patients who were non-responders at month 3 were advanced to tofacitinib 5 mg BID)	440[c]	69[c]	24 weeks	Dose-ranging study; tofacitinib combination therapy
NCT00550446 [3]	IIb	Active RA with inadequate response or intolerance to ≥ 1 DMARD	1, 3, 5, 10, or 15 mg BID monotherapy (patients who received 1 or 3 mg BID who were	Adalimumab 40 mg SC Q2W (switched to tofacitinib 5 mg BID at month 3); placebo (patients receiving	272[b]	112[b]	24 weeks	Dose-ranging study; active comparator; tofacitinib monotherapy

			non-responders at month 3 were advanced to 5 mg BID)	placebo who were non-responders at month 3 were advanced to tofacitinib 5 mg BID)				
NCT00603512 [4]	II	Active RA with inadequate response to MTX	1, 3, 5, or 10 mg BID with background MTX	Placebo with background MTX	112[c]	28[c]	12 weeks	Dose-ranging study; tofacitinib combination therapy; Asian patient population
NCT00687193 [5]	II	Active RA with inadequate response to ≥ 1 synthetic DMARD or bDMARD	1, 3, 5, 10, or 15 mg BID monotherapy	Placebo	265[c]	53[c]	12 weeks	Dose-ranging study; tofacitinib monotherapy; Asian patient population
NCT00976599[a] [6]	IIa	Active RA with inadequate response to MTX	10 mg BID with background MTX	Placebo with background MTX	15[c]	14[c]	4 weeks	Effect of tofacitinib on synovial pathobiology; tofacitinib combination therapy; US patient population
NCT01359150[a] [7]	II	Active RA not previously treated with tofacitinib	10 mg BID monotherapy or with background MTX	Placebo alone or with background MTX	100[c]	100[c]	9 weeks	Effect of tofacitinib on pneumococcal and influenza vaccine responses; tofacitinib combination therapy; US and

								Polish patient population
NCT00847613 (ORAL Scan) [8]	III	Active RA with inadequate response to MTX	5 or 10 mg BID with background MTX	Placebo (advanced to tofacitinib 5 or 10 mg BID at month 3 [non-responders] or month 6 [remaining patients]) with background MTX	637[b]	160[b]	24 months	Effect of tofacitinib on radiographic outcomes; tofacitinib combination therapy
NCT00814307 (ORAL Solo) [9]	III	Active RA with inadequate response or intolerance to ≥ 1 bDMARD or non-bDMARD	5 or 10 mg BID monotherapy	Placebo (advanced to tofacitinib 5 or 10 mg BID at month 3)	488[b]	122[b]	6 months	Tofacitinib monotherapy; inadequate response to bDMARD or non-bDMARD
NCT00853385 (ORAL Standard) [10]	III	Active RA with inadequate response to MTX	5 or 10 mg BID with background MTX	Adalimumab 40 mg SC Q2W; placebo (advanced to tofacitinib 5 or 10 mg BID at month 3 [non-responders] or month 6 [remaining patients]) with	405[b]	312[b]	12 months	Tofacitinib combination therapy; inadequate response to MTX; active comparator

				background MTX				
NCT00960440 (ORAL Step) [11]	III	Active RA with inadequate response or intolerance to ≥ 1 TNFi	5 or 10 mg BID with background MTX	Placebo (advanced to tofacitinib 5 or 10 mg BID at month 3) with background MTX	267[c]	132[c]	6 months	Tofacitinib combination therapy; inadequate response to TNFi
NCT01039688 (ORAL Start) [12]	III	Active RA not previously treated with MTX	5 or 10 mg BID monotherapy	MTX monotherapy (starting dose of 10 mg per week, increased by 5 mg per week every 4 weeks to 20 mg per week at week 8)	770[b]	186[b]	24 months	Tofacitinib monotherapy; first line
NCT00856544 (ORAL Sync) [13]	III	Active RA with inadequate response to ≥ 1 bDMARD or non-bDMARD	5 or 10 mg BID with background non-bDMARD	Placebo (advanced to tofacitinib 5 or 10 mg BID at month 3 [non-responders] or month 6 [remaining patients]) with background non-bDMARD	636[b]	159[b]	12 months	Tofacitinib combination therapy; inadequate response to bDMARD or non-bDMARD

[a]Trial was only included in safety analyses as its duration was less than that required for the efficacy analyses (i.e., 3 months)

[b]Randomized and received ≥ 1 dose of treatment

[c]Randomized to a treatment group

Abbreviations: bDMARD biologic disease-modifying antirheumatic drug, *BID* twice daily, *DMARD* disease-modifying antirheumatic drug, *MTX* methotrexate, *Q2W* once every 2 weeks, *QD* once daily, *RA* rheumatoid arthritis, *SC* subcutaneous, *TNFi* tumor necrosis factor inhibitors

References

1. Kremer JM, Bloom BJ, Breedveld FC, Coombs JH, Fletcher MP, Gruben D, et al. The safety and efficacy of a JAK inhibitor in patients with active rheumatoid arthritis: results of a double-blind, placebo-controlled phase IIa trial of three dosage levels of CP-690,550 versus placebo. Arthritis Rheum. 2009;60:1895–905.

2. Kremer JM, Cohen S, Wilkinson BE, Connell CA, French JL, Gomez-Reino J, et al. A phase IIb dose-ranging study of the oral JAK inhibitor tofacitinib (CP-690,550) versus placebo in combination with background methotrexate in patients with active rheumatoid arthritis and an inadequate response to methotrexate alone. Arthritis Rheum. 2012;64:970–81.

3. Fleischmann R, Cutolo M, Genovese MC, Lee EB, Kanik KS, Sadis S, et al. Phase IIb dose-ranging study of the oral JAK inhibitor tofacitinib (CP-690,550) or adalimumab monotherapy versus placebo in patients with active rheumatoid arthritis with an inadequate response to disease-modifying antirheumatic drugs. Arthritis Rheum. 2012;64:617–29.

4. Tanaka Y, Suzuki M, Nakamura H, Toyoizumi S, Zwillich SH, Tofacitinib Study Investigators. Phase II study of tofacitinib (CP-690,550) combined with methotrexate in patients with rheumatoid arthritis and an inadequate response to methotrexate. Arthritis Care Res (Hoboken). 2011;63:1150–8.

5. Tanaka Y, Takeuchi T, Yamanaka H, Nakamura H, Toyoizumi S, Zwillich S. Efficacy and safety of tofacitinib as monotherapy in Japanese patients with active

rheumatoid arthritis: a 12-week, randomized, phase 2 study. Mod Rheumatol. 2015;25:514–21.

6. Boyle DL, Soma K, Hodge J, Kavanaugh A, Mandel D, Mease P, et al. The JAK inhibitor tofacitinib suppresses synovial JAK1-STAT signalling in rheumatoid arthritis. Ann Rheum Dis. 2015;74:1311–6.

7. Winthrop KL, Silverfield J, Racewicz A, Neal J, Lee EB, Hrycaj P, et al. The effect of tofacitinib on pneumococcal and influenza vaccine responses in rheumatoid arthritis. Ann Rheum Dis. 2016;75:687–95.

8. van der Heijde D, Strand V, Tanaka Y, Keystone E, Kremer J, Zerbini CAF, et al. Tofacitinib in combination with methotrexate in patients with rheumatoid arthritis: clinical efficacy, radiographic, and safety outcomes from a twenty-four-month, phase III study. Arthritis Rheumatol. 2019;71:878–91.

9. Fleischmann R, Kremer J, Cush J, Schulze-Koops H, Connell CA, Bradley JD, et al. Placebo-controlled trial of tofacitinib monotherapy in rheumatoid arthritis. N Engl J Med. 2012;367:495–507.

10. van Vollenhoven RF, Fleischmann R, Cohen S, Lee EB, García Meijide JA, Wagner S, et al. Tofacitinib or adalimumab versus placebo in rheumatoid arthritis. N Engl J Med. 2012;367:508–19.

11. Burmester GR, Blanco R, Charles-Schoeman C, Wollenhaupt J, Zerbini C, Benda B, et al. Tofacitinib (CP-690,550) in combination with methotrexate in patients with active rheumatoid arthritis with an inadequate response to tumour necrosis factor inhibitors: a randomised phase 3 trial. Lancet. 2013;381:451–60.

12. Lee EB, Fleischmann R, Hall S, Wilkinson B, Bradley J, Gruben D, et al. Tofacitinib versus methotrexate in rheumatoid arthritis. N Engl J Med. 2014;370:2377–86.

13. Kremer J, Li Z-G, Hall S, Fleischmann R, Genovese M, Martin-Mola E, et al. Tofacitinib in combination with nonbiologic disease-modifying antirheumatic drugs in patients with active rheumatoid arthritis: a randomized trial. Ann Intern Med. 2013;159:253–61.
