# Supplementary material for: Efficacy and safety of tofacitinib in patients with rheumatoid arthritis by previous treatment: post hoc analysis of phase II/III trials
Source: Arthritis Res Ther. 2023 Nov 2;25:214. doi: 10.1186/s13075-023-03154-z (PMC10621211; doi:10.1186/s13075-023-03154-z)
Supplement: Supplementary file 2 — Additional file 2: Supplemental Fig. 1. fficacy outcomes in the bDMARD-IR population stratified by 1 or ≥ 2 prior bDMARD failure as assessed by proportion (95% CI) of patients achieving (A) ACR20, (B) ACR50, and (C) ACR70 response, (D) DAS28-4(ESR)-defined remission (score < 2.6), and (E) LS mean (SE) change from baseline in HAQ-DI score at month 3. [file 13075_2023_3154_MOESM2_ESM.pdf]

Supplemental Fig. 1 Efficacy outcomes in the bDMARD-IR population stratified by 1 or ≥ 2 prior bDMARD failure as assessed by proportion (95% CI) of patients achieving (A) ACR20[a], (B) ACR50[a], and (C) ACR70[a] response, (D) DAS28-4(ESR)-defined remission (score < 2.6)[b], and (E) LS mean (SE) change from baseline in HAQ-DI score[c] at month 3.

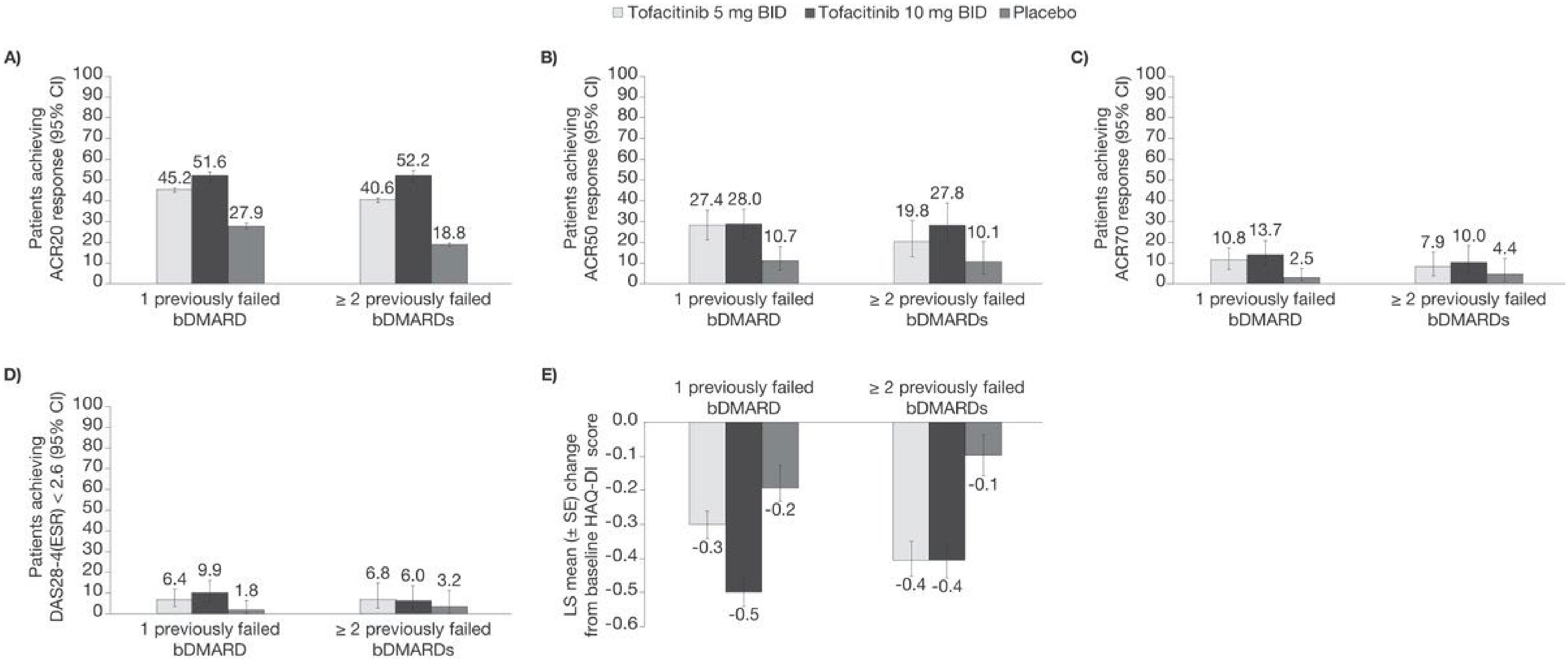

Data presented for the FAS; non-responder imputation (proportion of patients). [a]1 prior bDMARD failure/≥ 2 prior bDMARD failures: tofacitinib 5 mg BID, *n*=157/101; tofacitinib 10 mg BID, *n*=161/90; placebo, *n*=122/69. [b]1 prior bDMARD failure/≥ 2 prior bDMARD failures: tofacitinib 5 mg BID, *n*=141/88; tofacitinib 10 mg BID, *n*=142/83; placebo, *n*=113/62. [c]1 prior bDMARD failure/≥ 2 prior bDMARD failures: tofacitinib 5 mg BID, *n*=145/91; tofacitinib 10 mg BID, *n*=148/82; placebo, *n*=107/62. *ACR20/50/70* American College of Rheumatology ≥ 20/50/70% response criteria, *bDMARD* biologic disease-modifying antirheumatic drug, *BID* twice daily, *CI* confidence interval, *DAS28-4(ESR)* Disease Activity Score in 28 joints derived from 4 measures, erythrocyte sedimentation rate, *DMARD* disease-modifying antirheumatic drug, *FAS* full analysis set, *HAQ-DI* Health Assessment Questionnaire-Disability Index, *IR* inadequate response or intolerance, *LS* least squares, *SE* standard error
